# A Democracy-Friendly Theory of the Rule of Law

**DOI:** 10.1007/s40803-024-00240-5

**Published:** 2024-08-19

**Authors:** Jeremy Webber

**Affiliations:** 1 Professor Emeritus, University of Victoria, Victoria, Canada; 2 Honorary Professorial Fellow, University of Melbourne, Parkville, Australia

## Abstract

The dominant way of thinking about the rule of law is that it is a constraint, a limit, on government. On this view the limitation applies with full force to all forms of government, democratic and undemocratic, and to both the executive and the legislative branches. The privileged institution for enforcing those limits is the courts. Democracy and the rule of law are, in effect, portrayed as though they were in opposition to one another. That, I claim, is a mistake (a) historically (for, in the Anglo-American tradition, the rule of law developed first as a restriction on an undemocratic executive, with a less undemocratic Parliament acting in concert with the courts to institute the rule of law); (b) in principle (for there is a strong argument that democracy needs the rule of law for its fullest expression, and the rule of law needs democracy); and (c) strategically (because it hinders us from mobilizing our full resources to protect both principles; this paper began its life as a response to populist movements, many of which, wrongly, are conceded to be democratic). In this paper I make that case, especially focusing upon its most controversial claim, namely that the rule of law needs democracy. This paper forms part of a larger project on democratic constitutionalism in which I reconsider key concepts in constitutionalism in a manner that takes democratic decision-making to be fundamental to contemporary constitutionalism.

## Introduction

The dominant way of thinking about the rule of law is that it is, at its core, a constraint, a limit on government. On this view, the limitation applies with full force to all forms of government, democratic and undemocratic, and to both the executive and legislative branches. The privileged institution for enforcing these limits is the courts. Democracy and the rule of law are therefore portrayed as though they were set against one another, each identified with its institutional champion: democracy with the legislature, the rule of law with the courts.

This is, I argue, an oversimplification—such a simplification that it impairs our ability to pursue both democracy and the rule of law as effectively as we might. It treats democracy and the rule of law as being in opposition when they are, for the most part, coordinate and interdependent. It leads us to neglect the ways in which both principles can be, indeed ought to be, pursued in tandem. It encourages us to treat the courts as the unique guardians of the rule of law, neglecting the ways in which the democratic branches of government, if well designed and functioning, also advance that value. The oversimplification fosters a bias against democratic action—a bias in which respect for rights is accomplished, quintessentially, by limiting democracies. The democratic state is treated as the enemy of rights, not the primary vehicle by which rights have been achieved.

The oversimplification has an especially pernicious impact when it comes to constitutional lawyers’ criticisms of populist regimes.^1^ Constitutional lawyers often criticize such regimes, above all, for failing to observe the limits imposed by the rule of law. However, in framing their criticisms solely in terms of limits, they can leave unchallenged populist governments’ claims to be acting democratically. They concede by default the populists’ formulation of the issue, in which advocates of the rule of law seek to restrict democracy, lawyers and courts seek to frustrate the people’s will. That formulation profoundly mischaracterizes the situation. Those populists who deserve criticism—here I speak only of those who deserve criticism for the populist label is sometimes applied to politicians who are merely boisterous democrats—are generally not deficient merely in their respect for law. They are also bad democrats. They work hard *not* to let the people govern by (for example) stifling the mechanisms by which the citizenry can debate the future of their country, constraining citizens’ access to information about what their governments are doing, undermining the institutions through which the people choose their representatives, limiting discussion in parliament, corrupting the mechanisms by which laws adopted by the people’s representatives are enforced, and undermining the structures that compel public officials to execute their duties and employ public funds only for the people’s purposes, not for their private benefit. The papers in this special issue provide examples in relation to the countries of Central and Eastern Europe (CEE). János Mécs’s paper specifically addresses elections, other papers in this issue deal with important aspects of democratic governance, and there are many additional examples in what is now a voluminous literature (see, as a very partial list, von Bogdandy and Sonnevend 2015; Pap 2018; Bugarič 2019; Sadurski 2019, 2022; Drinóczi and Bień-Kacała 2021; Krygier et al. 2022). Authoritarian populist regimes ought to be criticized in democratic terms, directly rebutting populists’ primary claims to justification. Their deficiencies in democratic practice are interdependent with their deficiencies with respect to the rule of law. The two failings go hand in hand.

Conversely, when populists are acting democratically, their actions and their arguments deserve careful consideration even if one disagrees with them. Populists may be drawing our attention to defects in our democratic institutions, frequently to the lack of responsiveness of the institutions to matters of public concern, or to the impact of inequality (for left populists, including economic inequality) on public policy. Indeed, even when populists are acting undemocratically, they may, despite themselves, prompt us to recognize defects in our current democratic orders. They may be exploiting widespread dissatisfaction with institutions that have come to be seen as out of touch. The best response may be the renovation and re-justification of our democratic institutions: the intensification of democracy, not its limitation.

My principal purpose in this paper is not to document the undemocratic practices of many populist regimes. For that, I am content to draw upon the work of others, especially the authors of the fine contributions to this special issue. Rather, I seek to correct the oversimplification that often distorts and diverts the debate: the idea that the rule of law is opposed to democracy—that it operates primarily as a limit upon democracy. Here I advance a democracy-friendly understanding of the rule of law—one in which democracy and the rule of law are understood to be interdependent and mutually reinforcing, each tending to foster and complete the other.

In doing so, I reject a strategy employed by some lawyers and legal scholars—that of expanding the concept of “democracy” so that it includes, by definitional fiat, the rule of law. Indeed, often the definitional inflation doesn’t end there; the rule of law itself is taken to include a broad array of court-interpreted and court-protected human rights.^2^ This inflation is unfortunate for it obscures the relationship among institutions, providing few resources for analyzing, let alone managing, the tensions that can arise among them. Moreover, such a strategy is self-defeating. Precisely because it relies upon definitional assertion, anyone who isn’t already convinced will be left unpersuaded. Indeed, such a claim can reinforce, not dispel, populists’ assertions that the people are not being permitted to govern. I will therefore adopt deliberately pared-down definitions of both democracy and the rule of law—definitions that focus on the distinctive core of each concept.

Focusing on that core does two things. First, it widens the scope of those to whom I hope the argument will prove convincing. The definitions of both democracy and the rule of law are famously contested – “essentially contested” in the sense developed by Gallie (1955) in which the concepts’ very value lies in continuous debates over what the concepts ought to require (Waldron 2021; Webber 2023: 851–2). Those debates are nevertheless driven forward by aspirations that are, in very general terms, broadly shared. It is that general orientation I seek to capture in each of my pared-down definitions. By relying upon general aspirations, I hope that my arguments prove convincing to people who may disagree over the concepts’ further implications. Such an approach also forces anyone who rejects the pared-down definition to offer their own alternative, allowing an observer to judge whether that alternative conforms to their impression of what the concepts are all about.

Secondly, such pared-down definitions invite, rather than foreclose, arguments over the institutionalization of the aspirations. Those further arguments are crucial for any real-world responses. The pared-down definitions are not meant to take the place of those extensive arguments but rather to help frame and guide them. Often inflated definitions do foreclose such arguments. They take an extensive institutional structure for granted. They attempt to roll a whole panoply of institutional consequences into the definitions themselves. But the institutional consequences are difficult and contestable. They deserve our concerted consideration. This is so especially (but not exclusively) when we are responding to the rise of populism. I want to invite those questions, not foreclose them. Moreover (to put all my cards on the table) I do so in part because I believe that there is no substitute for decision-making by the people’s representatives—for democratic justification, not substantive norms interpreted by courts—as the foundational principle of contemporary constitutionalism (Webber 2006b). I accept that a well-ordered society requires that there be an institutional separation between judges and elected officials (Webber 2006a: 275–7). Such institutional arrangements have generally been instituted by democracies themselves. I also accept that such a society requires respect for certain human rights. Indeed, establishing the need for such protections is one of the principal objectives of this paper. However, in my view that need flows from—it does not contradict—the principle that political and legal institutions ought to be arranged so that the people are responsible for their own governance. If one accepts that principle, one ought to demand strong justification before one hives off matters for resolution by institutions that escape democratic control. Nevertheless, to be clear, I will not be asking readers to accept my full position in this paper—merely that those arguments—to my mind essential—not be foreclosed.

What then are the pared-down definitions of democracy and the rule of law?

For democracy, it is the principle that citizens are entitled, collectively, to govern themselves. This principle operates at a broad level of generality. It can be advanced through a wide range of institutional forms: representative, direct, and deliberative. This definition is, then, open as to the choice of mechanisms, including the substantially different forms used in non-state societies.^3^ It does, however, insist upon mechanisms that enable the practical and effectual participation of the citizenry—of flesh-and-blood people, not the attributed voice of an imagined people. It rejects, then, some theorists’ disdain for mechanism and their insistence upon a theorist-determined or strong-man-inspired “substantive” democracy (see Carl Schmitt's position of this kind, McCormick 1997: 167–75, 240–2). It specifically rejects the claim of authoritarian populists to speak for a supposed people, a people that those same populists often do their best to disempower. Citizens and theorists may—indeed they ought to—argue over the adequacy of the mechanisms. But for the purposes of this paper it is sufficient to say that measures that advance democracy are measures that enhance the capacity of citizens to participate in, and see as their own, the collective decisions that govern their society.

This emphasis on participation also means that I will be focusing primarily on large assemblies (in states, the legislature) rather than the executive as the institutional embodiment of the democratic principle. There is good reason to think that the rule of law applies more stringently to the executive than to the legislature. This is sometimes said to be because the executive controls the day-to-day instruments of coercion, which is true. That fact is supplemented by a representational difference. The executive, almost always, represents a narrower segment of the political spectrum than does the legislature. The executive includes, internally, substantially less of the plurality of positions that exist within society at large. That broader plurality does not contribute to the framing of executive initiatives, nor is it present to monitor, question, and criticize during policy formation. As a result, the discursive controls are less salient. It will not be possible, in the space of this paper, to explore those differences fully (see, for a first attempt, Webber 2008). But one should guard against the elision that often occurs between the executive and the legislature in the rule-of-law literature.

For the pared-down definition of the rule of law, I focus on the virtues associated with the governance of society through law—through, that is, the use of general rules, capable of being known by citizens in advance, so that citizens have the opportunity to organize their affairs within the framework provided by those rules. This definition concentrates on the distinctive form of law rather than on the purposes pursued through law. It emphasizes the value of using law as the primary instrument for relations between the state and the people regardless of the specific ends that governments pursue.

This focus on form rather than substance is often denigrated in today’s rule of law literature, which generally dismisses the focus on form as merely rule *by* law.^4^ That objection is, I think, unjustified—the result of a failure to take questions of form seriously enough. The aspiration to use general rules, known in advance, supports an important conception of the relationship between government and citizen. Lon Fuller (1969) called this conception the law’s “internal morality” and distinguished it from law’s “external morality”—from those additional moral purposes that are not themselves inherent in the legal form but that governments or citizens may seek to achieve through law. Fuller didn’t object to the pursuit of an external morality. On the contrary, he strongly supported some version of it, including respect for fundamental rights. But he also wanted his readers to recognize the particular value of law’s form so that institutions would be employed to sustain and advance that form’s distinctive value. He expressed the key features of the internal morality through his “eight kinds of legal excellence toward which a system of rules may strive” (Fuller 1969: 41). They are, to adopt the words of the foremost interpreter of Fuller, Kristen Rundle (2013: 2; Fuller 1969: 38–9): “generality, promulgation, clarity, avoidance of contradiction and of impossibility, constancy through time, non-retroactivity, and the requirement that there be congruence between official action and declared rule.” These eight qualities capture the rule of law’s central concern with preventing arbitrary punishment, subjecting the state to the discipline of law. They do so in the interest of sustaining what Rundle (2013: 89–92, 97–101) describes as a relationship of respect and fidelity between lawgivers and citizens, where the latter are addressed as responsible agents, bearers of dignity. Fuller’s eight criteria have been widely accepted as indicia of the rule of law. I will follow that practice in this paper.

In adopting this definition, I entirely reject one characterization of the rule of law, namely that the rule of law means that the law itself rules. On this view, the substance of the law should not be specified by citizens, legislature, executive, and courts (as in Fuller’s theory) but by principles inherent in the law itself. It is sometimes expressed as an aspiration to achieve “impersonal law” in which legal principles—principles of right reason—abstracted from the purposes of citizens or officials, furnish an ideal framework within which individuals can pursue their own ends. James Harrington’s evocative phrase—“a government of laws and not of men” (Harrington 1992 [1656]: 35)—is sometimes harnessed in support of this vision. It typically contemplates a strictly limited role for government and protects, above all, rights of property. I will address one of the most developed arguments along these lines (that of Michael Oakeshott) in Sect. 3.3.1 below but, for the moment, suffice it to say that my principal reason for rejecting this position is that law, as a human creation, is never divorced from human purposes. It always has human authors, and those authors always define the law against a backdrop of diversity and disagreement. That is true even of the small-state, property-based libertarianism of Michael Oakeshott or Friedrich Hayek, as we will see. Indeed, it is ironic that Harrington’s words are cited in support of a disembodied impersonal law. To achieve the best approximation to divine reason, Harrington proposed what was very much a human institution: a novel, bicameral, representative parliament, underpinned by rough equivalence in the material resources available to each of the political classes. For him, it was debate and decision by such a legislature, followed by faithful enforcement by the executive, that best exemplified the government of laws (Harrington 1992 [1656]: 10–25; see also Pocock 1992: xxi–xxiv; Hammersley 2019: 109–21 on Harrington as a democrat).

Neither of my pared-down definitions of democracy and the rule of law contains a list of constitutionally-entrenched rights enforced by courts. Such a list is included within many accounts of the rule of law and certain rights (notably freedom of speech) are typically read into the definition of democracy. I will resist incorporating them into the definitions for two reasons. First, those who include those rights often fail to address how the rights are to be secured. Worse, they often assume without argument that any rights will be defined and enforced by courts. But the questions of how rights are to be developed are not trivial. While many accept the importance of those rights in general—I do myself—their definition and their balancing against competing interests are subject to reasonable disagreement (Waldron 1999: 211–31). Should, for example, corporate advertising fall within freedom of speech, so that regulation needs to be justified under demanding constitutional standards? Should governments be able to regulate election spending in order to promote equality of participation in political debates? Should advocacy of violence qualify as protected speech, and how should one define what counts as violence? Given disagreement over such matters, there is a strong argument that the legislature ought to share in determining these matters. At the very least, the question of institutional role needs to be addressed directly, not hidden within a broad definition of democracy or the rule of law (see Webber 2006a).

Second, the panoply of rights protects a wide range of interests—not merely those related to democracy and the rule of law—and yet expansive definitions of the terms rarely specify the particular rights that should be included. On the contrary, they generally presume that those principles include a full catalogue of rights. Again analytical clarity is sacrificed and, when a long list is combined with the assumption that judges are the unique interpreters of rights, the question of which institution is best placed to develop the rights is neglected once again. This is especially unfortunate once one realizes that some important rights—rights to housing, health care, education, or a decent income—have historically depended entirely upon legislative action. Again, questions of institutional role should be addressed directly, not obscured by our definitions.

To be clear, I am not arguing that there are no connections between rights, democracy, and the rule of law. There are, and those connections will figure prominently in this paper’s argument. Freedom of speech, freedom of association, equality, rights to education, limitations on hours of work, and other rights are, for example, fundamental to democratic participation. Moreover, the entire purpose of this paper emphasizes the interdependence of the concepts of democracy and the rule of law. It seeks to unpack the insight, expressed by theorists as diverse as Jürgen Habermas (1996: 408–10, 449–50) and James Tully (2008b: 92, 162), that democracy and the rule of law are “co-original” or “equi-primordial” in contemporary constitutionalism.^5^ I suspect that many thinkers who invoke this insight still think of the two principles as being in implicit opposition, with the rule of law constraining democracy. I argue that the two principles are co-original in a more fundamental sense, supporting and enabling each other, so that the full realization of each can be achieved only in the presence of both.

This paper is structured around that interdependence of the principles. Its first half argues that democracy needs the rule of law. It explores three ways in which this is so: (1) the rule of law supports the exercise of freedoms essential to democratic participation; (2) the rule of law enables citizens to make collective decisions despite the disagreement that exists within any human community; (3) the rule of law helps to ensure that, once made, those decisions maintain their integrity through to the point of implementation. The proposition that democracy needs the rule of law will, I suspect, be relatively uncontroversial. Some of its features will be familiar from the work of others. But it is worth exploring to guard against some of the claims made in the name of radical and revolutionary democracy—claims that have surprising echoes in authoritarian populist movements.

The second half will argue that the rule of law needs democracy. This branch of the argument is more controversial. It is common in the rule of law literature to suggest that the rule of law can exist in the absence of democratic government, indeed that it arose historically in undemocratic societies. Such an account is, at the very least, exaggerated. I don’t deny that some rudiments of the rule of law can exist in undemocratic societies but when they do the rule of law tends to be anemic and fragile. Any robust instantiation of the principle depends upon features of an emphatically democratic politics. This association is not merely circumstantial. It is integral. Democratic decision-making is the best way of securing central features of the rule of law.

Both these arguments depend upon seeing democracy and the rule of law as complex social attainments, the achievement of which requires experience, engagement, reflection, and judgement. The principles are “kinds of excellence”, to adopt the term that Fuller (1969: 41) used to describe the features of the rule of law. They are attained by degrees. They are inevitably works in progress. And they are most highly developed when pursued together. The relationship between the principles can be tendentious at times, but neither principle is the enemy of the other. They are each the condition of the other’s better attainment.

That realization ought to shape how we pursue both principles. The limitation of legislative activity by courts is not the dominating feature of their relationship. The better achievement of democratic self-government, exercised through forms that respect the agency and responsibility of citizens, is. There may be times when those forms impose some constraint on the actions of legislatures—there certainly will be times when they constrain the executive—but then again, there may be times when courts too ought to be constrained by legislatures in the interest of the rule of law. Think, for example, of the current proposal that judges of the US Supreme Court should be required to comply with basic principles of judicial ethics (Murphy and Berg 2023). The fundamental point is that our analyses are distorted if we presume that the rule of law is primarily concerned with limiting democratic legislatures. On the contrary, the rule of law is best achieved through those institutions’ constructive, robust, interaction.

In one of his illuminating allegories, Lon Fuller (1968: 105–06) imagines a tyrant who wants to maximize his entirely selfish interests. The tyrant soon realizes that he must allow some freedom of action to his subjects if he is to secure his interests most effectively. It is costly to direct his subjects’ actions in every detail and to supervise them closely to ensure they comply. It is much more efficient to establish general guidelines, which the subjects are then empowered to secure in their own ways. Once he does so, however, he finds that he too must consider himself bound by the structure he has established. Otherwise, his subjects will realize that the rules are a charade, and all benefit of the tyrant’s strategy will be lost. Thus we see the emergence of some rudimentary version of the rule of law (Fuller 1958: 344; 1969: 38–40; compare the similar account of Stephen Holmes 1995: 113–20, in his discussion of Jean Bodin on the exercise of sovereignty by absolute monarchs). But interestingly, Fuller does not stop his allegory there. His tyrant now realizes that “a human being will serve as a more effective tool if he is happy and satisfied with this role” and that this happiness will be more effectively achieved if the subject is able to serve “not only the ends of another, but his own as well” (1968: 106).^6^ Thus the erstwhile tyrant no longer requires that the subjects serve only the tyrant’s interests; he also permits them to serve their own. Note that it would not take a great leap to extend the allegory yet again to include the ruler’s creation of some institution for consulting a representative section of his subjects. Some such mechanism would be useful to ascertain the subjects’ desires and gauge their level of satisfaction. And once such a representative council is created, those representatives might well occupy, in effect, a role in the ruler’s decision-making. Such an evolution is familiar from the historical emergence of conciliar and ultimately parliamentary government. Of course, such a favourable evolution is not guaranteed. A tyrant might realize that he is on just such a path and act to forestall it. If he does, that might mean rolling back protections already granted, including those approximating the rule of law. The current developments with respect to the rule of law in China are an example of such a reversal. But one nevertheless sees, in the development of Fuller’s allegory, the affinity that exists between the rule of law and popular participation in government. The source of that affinity lies in basic decision to recognize and work with the agency and responsibility of citizens.

And finally, one last introductory comment: I hope that readers will see, throughout this paper, observations that harmonize with the work of other scholars on democracy and the rule of law—connections, alas, that won’t always be express. Two factors account for the incompleteness of the references. First, the literature on these topics is now so vast that it is impossible, even in a paper of this length, to discuss the literature comprehensively. Second, much of that literature is framed in relation to specific constitutional contexts and makes more concessions to those contexts than I wish to make in this general argument. The rule of law is a chameleon (to adopt an image that Stambulski, in his contribution to this special issue, applies to populism); it incorporates features from its institutional backdrop, presenting differently in the United Kingdom, for example, given parliamentary sovereignty, than it does in the United States, given that country's written constitution and entrenched bill of rights. Lessons learned from one context often require considerable adjustment before they can be applied to another—explanations that have rarely been possible within the limitations of this paper. I hope that those friends from whom I have learned so much will find more generous acknowledgements of their work in my other writings.

## Democracy Needs the Rule of Law

It is common to focus upon contested elections as the essence of democracy, but that is far too narrow. Elections certainly are important features of contemporary state-structured democracies, but even in states they are not the sole vehicle for citizens’ engagement in governance. Citizens also make decisions directly through referenda and shape public policy through advocacy and consultation. The decision-making structures of non-state societies are even more diverse. Here, I focus on the principal challenge for all democratic decision-making, whatever form it takes, namely how to move from the multitude of voices in any society to an authoritative expression of the society’s position—from the diversity of citizens’ actual voices to a more focused (though provisional) public voice.

That public voice is constructed. It abstracts from the multiplicity of the citizens’ actual voices. It is, in that sense, artificial; it may not accord in its particulars with the voice of any one individual. But the mechanisms for decision in a well-ordered democracy produce a sifting and aggregation of opinion that result in a conclusion that a critical mass of citizens consider to be the legitimate expression of the society’s position. That of course opens up wide scope for argument: what constitutes a “critical mass”? What makes a democracy “well-ordered”? Who should be considered “citizens”?^7^ How are their opinions expressed and aggregated? Those questions are precisely what furnishes room for debate over citizenship, participation, electoral systems, modes of representation, rules for decision, the list goes on. Those debates matter. They engage fundamental values of equality and self-determination. They form the lynch-pin between the people and the decisions adopted in the people’s name. They are precisely why democracy is an “essentially contested concept” (Gallie 1955: 183–7). But note three things. First, the ultimate “artificiality” of a collective voice cannot be avoided. The voices within society *are* irredeemably diverse. We can hope to achieve a measure of concord; we can create conditions that foster that possibility; but our reach will always exceed our grasp. The outcome will always be some aggregation of the disparate voices within society. Note, however, that such artificiality is not confined to democracies. Authoritarians everywhere—including authoritarian populists—claim to speak for a unified people without institutional mediation. That claim is always a lie. It is only sustained to the extent that the actual people, with their many voices, are discouraged from speaking or are excluded from the ranks of the people. The use of institutional means to construct a societal position is not a defect of democracy. It is democracy’s great achievement. Institutions are foundational to any genuine self-rule (Holmes 1995: 167).

Second, note that, even in healthy democracies, disagreement extends to the institutional structure, indeed often to such foundational elements of that structure as the electoral system, the definition of electoral boundaries, accession to citizenship, and the use of direct democracy. It is not the case, as some theorists claim, that democratic government, to be legitimate, requires that citizens agree to the society’s governing institutions. Full agreement is an unattainable standard. What is required is that there be widespread acceptance that collective decision-making must occur through some institutional structure and that the current institutions are better than none—even if one seeks, with all one’s might, to amend or replace them. Even in a healthy democratic order, citizens will recognize that they disagree over the institutional structure just as they do over many things, but they also recognize that it is essential to have some such structure for making collective decisions. This isn’t an argument for quietism. We ought to hope for, strive for, institutions more worthy of members’ full consent. But we also realize that a society’s institutions are framed within, not outside, history. They are the product of an ongoing political process. Citizens will disagree over the merits of their institutions. That may mean that we must put up with institutions that we ourselves consider to be imperfect.

Third, note that the society’s voice on any of these matters is, in principle, provisional, not permanent. The people are entitled to participate in their society’s collective decisions not just on one occasion but for all time. They remain sovereign, through their institutions, at every moment. They must be able to change their minds, to learn, to persuade their compatriots to change, and to benefit from that persuasion. A democracy is (to borrow a phrase Renan (1882: 27) used in a different sense) a “plébiscite de tous les jours”. The definition of the common good is a continual work in progress.

When looking, then, at the relationship between democracy and the rule of law, it is worth examining three processes in this endeavour of collective self-rule: (1) the discussion and debate that occurs within society at large through which the society’s members learn, formulate positions, persuade others, and change their own minds; (2) the institutional mechanisms through which the society fastens upon an outcome as the society’s position on that matter (albeit provisionally); and (3) the processes by which the society’s position is implemented. As we will see, the rule of law is essential to each of these dimensions. In the case of (2) and (3) the rule of law is utterly integral: one cannot achieve a democratic outcome without some ethic of fidelity to law. In the case of (1), the rule of law is perhaps not essential in quite the same way, but it is nevertheless crucial in setting the conditions for free disagreement and debate.

### The Rule of Law and the Democratic Freedoms

To begin with the first dimension, the development of an informed, engaged, and politically effective public depends upon certain rights and freedoms, most obviously freedoms of speech, the press, association, and assembly, but also including education, language rights, some degree of economic equality, and patterns of inclusion and exclusion generally within the society, all of which shape the extent and effectiveness of political participation.

All these rights are facilitated by the rule of law even in the narrow sense adopted in this paper. One cannot be secure in the exercise of rights if (a) one is substantially unsure about the standards of permissible conduct or (b) government officials routinely depart from the standards in practice. Seven of the eight excellences identified by Fuller as part of the internal morality of law seek to ensure these conditions. They are often undermined by today’s authoritarians, including authoritarian populists. Authoritarians do so both directly and indirectly: they may pay lip service to the people’s rights but subvert them by tolerating actions by officials that contravene the law; they may decline to enforce the law against private actors who intimidate the government’s opponents. Indeed, by taking an indirect approach, not only do authoritarians (including populist authoritarians) maintain a pretence of supporting democratic decision-making while undermining it in practice; they also sow insecurity and self-censorship among a populace unsure as to when and how repression might occur. Fidelity to law is essential to secure the rights and freedoms on which democracy depends.

### The Rule of Law and the Determination of the Society’s Decision

The rule of the law is even more central to the second and third dimensions identified above. The second dimension involves the determination of a single position to serve as the society’s decision on a given matter. That position is never in fact common to all citizens. It is always the construction of a common position justified by (a) the value of having *some* common position to govern the matter, and (b) the status of the constructed position as a defensible approximation of a common position, in which construction citizens have had an opportunity to participate.

The authority of the outcome is a function of both the quality of the procedure and the society’s compliance with it. In the short term, however, the assessment of the procedure tends to be rough and ready, essentially whether the bulk of the people accept the process as constituting a legitimate voice of the society, for citizens disagree over the merits of those procedures as they do over other matters. Think, for example, of the controversies over campaign financing; or the choice between first-past-the-post, proportional representation, and preferential voting; or the apportionment of constituencies—the list could be endlessly extended—all of which can have a profound effect on a democracy’s government. Thus, in the short-term assessment of quality, there is an inevitable bias towards accepting the society’s extant procedural structure not because that structure is better than other conceivable structures, but simply because the structure is known, citizens can anticipate how and when they ought to participate, they know how their participation will be aggregated, and, above all, the structure’s very givenness means that it is capable of providing definite outcomes. The sheer facticity of the process is the extant procedure’s greatest advantage. Without some settled process, there would be insufficient means to determine which assertions and counter-assertions should prevail, creating a significant risk that the outcome would be determined by whichever party could mobilize the greatest coercive force. The assessment of quality is thus, in the short term, generally reduced to an assessment of validity: Has the decision been made by the society’s established processes?^8^

To be clear, arguments over substantive assessments of quality remain important. They operate in at least three ways. First, the electoral system itself is subject to democratic determination and refinement. One of the great merits of democratic institutions is not that they always get the outcomes right—they don’t—but their capacity for reflexivity: the fact that they expose those institutions to criticism and change in a manner that is responsive to popular deliberation. There are, one might say, two time periods relevant to the assessment of quality: an immediate period, which governs the making of a specific decision, where the standard is validity; and a longer timeframe during which revisions are debated, decided, and implemented. Second, even given an ethic that focuses on validity, courts cannot help but draw upon substantive criteria in their interpretation of the law. Indeed, this is an important implication of Fuller’s theory (Fuller 1958; Rundle 2013: 168–74; see also Webber 2007). The determination of validity requires an ethic of fidelity to the decisions of the legislature, but interpretation is never merely mechanical.

Third, importantly, the existing process is always vulnerable to extra-legal change motivated by substantive concerns—to change that does not respect existing rules either to a limited degree (civil disobedience) or through wholesale rebellion and revolution. That possibility should never be forgotten. In situations in which democratic processes are absent, severely degraded, or exclusionary, the system can forfeit any claim to the respect of people subject to it. The development of democracy itself has been driven by such ructions. This was true of the English Civil War, the Glorious Revolution of 1688, and the American Revolution; the sustained struggles for decolonization in Asia and Africa; the Chartist, suffragette, and women’s equality movements; struggles for racial equality; Indigenous struggles; and the anti-Apartheid movement. And this is of course a partial list, neglecting entirely (among other things) attempts to secure democratic government on the European continent. An acute desire to take part in shaping one’s destiny, a commitment to equality, an awareness of exclusion, and the resulting civil unrest are sometimes indispensable spurs to change.

But here is the thing: reliance on extra-legal strategies alone, even for those driving change, is almost always second-best to change accomplished through established institutions. Indeed, extra-legal strategies are typically resorted to in circumscribed form, in parallel with institutional initiatives. They become the exclusive option only after existing institutions have failed comprehensively. Moreover, even when extra-legal strategies have been successfully pursued, the outcomes are often enacted using pre-existing institutional forms. This is true despite the fact that changes through existing institutions, even if successful, are likely to produce compromised outcomes, not complete victory. Why the reluctance to adopt purely extra-legal strategies? The answer is that extra-legal change sacrifices the advantages of a recognized process, making it difficult to fasten upon a single outcome and running the risk that that outcome will be left to whomever can marshal the most thugs. Indeed, the lack of process can impair members’ political agency even within a revolutionary movement. The members too require mechanisms for settling internal differences. And of course, defenders of the status quo and authoritarian movements often control the most resources, including the most effective means of coercion. Relying on existing processes secures at least some limitation on that use of force.

For all these reasons, even movements for democratic emancipation have a substantial interest in trying to make imperfect institutions work. Even when they go outside those institutions they generally do so to a limited degree, or they build upon existing models when framing their new institutions to gain some advantage from the established process, or they depend implicitly on the existing institutions remaining largely in place so that their opponents’ extra-legal action will be limited by the institutions’ staying-power. Such strategies were evident in the principle of a “self-limiting revolution” pursued by the revolutionary actors in the transition from communist rule in the CEE itself (Arato 1993: 674–77). Their societies had experienced the consequences of an unlimited revolution. The revolutionary leaders therefore saw the value of, for example, enacting constitutional amendments by the procedures stipulated in communist-era constitutions. All that said, more might have been done post-transition to embed the new institutions within thoroughly democratic processes. As we will see in Sect. 3.1, some have argued that that neglect paved the way for the current populist conjuncture.

Fidelity to law is therefore fundamental to the very possibility of democratic decision-making—to the capacity to fashion, by participatory means, a collective decision. That is certainly true in normal times, but even when people decide that the established procedures must be set aside in order to accomplish an indispensable change, they generally recognize that they should limit the extent of departures and seek, as soon as possible, to establish regular decision-making. Any genuinely democratic order, in which the citizenry participate in their own governance on a basis approaching equality, depends upon knowable processes of decision-making that are followed in practice. Democracy depends—not solely, but as one essential element in a set of achievements—upon the rule of law.

### The Rule of Law and the Implementation of Society’s Decisions

The same can be said, much more briefly, of the third dimension of collective self-rule: the implementation of the law. Democracy requires that the integrity of the law, democratically adopted, be respected right through to a law’s ultimate application. If that law is ignored, distorted by corruption, or undermined by government officials’ or judges’ lack of fidelity to good-faith interpretation of the law, then the people’s ability to rule themselves is sabotaged. In this respect too, fidelity to law is integral to democracy.

### Democracy Needs the Rule of Law: Final Comments

Fuller’s conception of the internal morality of law is based, as Rundle (2013) shows so well, on respect for citizens’ agency: respect, that is, for citizens’ actual capacity to know the legal regime that applies to them and to arrange their affairs in that knowledge. Fuller’s respect for citizens’ agency as the addressees of law bears a consonance, a symmetry, with the respect due citizens as the authors of law in a democracy. That symmetry fits well with a venerable theme in democratic theory: that the people who make the law ought to be bound by that law so that every citizen, even those directly engaged in government, is both an author and addressee of law.

It is little wonder, then, that disrespect for the people as authors of the law should, in authoritarian regimes of all varieties, be mirrored in disrespect for the people as addressees of law. Today’s authoritarians—including authoritarian populists—are loath to own that disrespect. They claim to support democracy but subvert it through the erosion of legal processes that facilitate participation: staffing electoral authorities and courts with appointees who will advance the authoritarians’ agenda; retaining such institutions but limiting their mandates; invoking emergency powers on spurious grounds in order to set aside normal legislative procedures; getting thugs to do their dirty work; intimidating NGOs; rushing laws into force with little debate. Authoritarians of every stripe generally make a show of acting in the people’s name but their actions are marked by disrespect for the citizenry both as addressees and as authors of law.

## The Rule of Law Needs Democracy

The argument thus far—that democracy requires the rule of law—is unlikely to meet much opposition from lawyers and legal scholars. The argument in this section—that the rule of law requires democracy—will be more controversial.

Most lawyers and legal academics speak as though the dependency only runs one way. The law, and especially the constitution, comes first. It establishes the foundation for legitimate government. Democratic structures are then built upon that foundation, with the law of the underlying constitution necessarily limiting what the government can do. One sees that vision in the potted histories told about the struggle to establish the rule of law. Those histories describe a series of efforts to constrain arbitrary government and subject it to legal limitations, the success of which brings into being the rule of law. The rule of law in those accounts is all about the imposition of limits.

But here is the problem. Those historical struggles were conducted against autocratic, not democratic governments, and in those struggles the advocates of the rule of law and the advocates of democracy were usually on the same side (it would be better to say democratization rather than democracy, for the political *demos* was, especially in the early stages, much less than complete). The extension of democracy was an integral part of the taming of arbitrary government. It was part of the answer, not part of the problem.^9^

It is of course possible that, following democratization, the new democracies might generate their own forms of arbitrary government for which the rule of law furnishes the answer. But the fact that their partisans have generally acted in alliance should alert us to the possibility that the two principles may be coordinate, supporting each other, enabling each other. The extension of the rule of law may in fact require more democracy, not less. In this section I explore why that is so—why the full achievement of the rule of law depends upon democratic institutions. I proceed in three steps. I begin with the most general way in which the rule of law is dependent on democracy: that any authority of the state, including the authority of legal institutions, is substantially dependent on democratic legitimation. Second, I explore how the central attribute of the rule of law—the faithful application of the law—is supported by an indispensable feature of democratic government: mechanisms of oversight, accountability, criticism, and debate. Third, I examine how certain elements of the rule of law—the aspiration towards generality in the law and the capacity of citizens to know what the law requires—are best achieved in conjunction with democratic institutions.

To be clear, I will not be arguing that every institution within a democratic order must do what the majority wants. There is often a tendency to drift towards all-or-nothing arguments: either the democratic populace is always right, or the insulated institutions of reasoned deliberation get it right (typically in this perspective the courts). That is not my approach. I support what was once the distinctive art of the constitutional lawyer: blending different institutional forms so that their strengths are maximized and their weaknesses minimized. It makes sense, for example, to protect judicial independence to ensure that the application of the law to particular cases is not distorted in a rush to achieve a general policy (Webber 2006a), and there are, of course, compelling arguments for insulating other institutions from direct majoritarian pressures (eg electoral commissions; redistricting processes; independent auditors of program costs; central banks). But the point remains that even such insulation must rest upon democratic legitimation and there is good reason for a democratic legislature to retain overall stewardship of a state’s legal order. Indeed, the blending of institutional forms is generally adopted by democratic legislatures themselves.

### Democracy and Support for the Institutions of the Rule of Law

In today’s world there is little alternative to democratic legitimation. All governments claim to govern with the people’s support. Even authoritarian regimes, even today’s monarchies, even theocracies, certainly authoritarian populists—claim to act on behalf of and with the support of their people. They assert a right of self-determination with that “self” being their people. They may display real anxiety as to whether they enjoy that support. Authoritarians of all stripes (including the Orbáns and the Kaczyńskis) try to monopolize the press and turn it into a vehicle for ruling-party propaganda, restrict NGOs, control universities, shield the executive from disclosure, intimidate their opponents, tamper with the machinery of elections, and drastically restrict the time permitted for parliamentary debate precisely because they fear that, if tested by fully democratic institutions, they would forfeit their pretence of majority support. But such efforts are a backhanded compliment to the power of popular legitimation. Even China’s leaders, despite their total control of the police and army, stifle political competition and rigorously police the information space precisely because they fear that their claim to the people’s support is fragile and, if tested, might collapse.

The requirement of popular legitimation also applies to the rule of law, including the authority of legal institutions. That is true, for example, of nations’ constitutions. Those constitutions are almost universally justified as an expression of their people’s will. That claim to legitimacy is frequently complex. Constitutional theorists often assume that more than majority support is required; indeed sometimes the consent of each and every citizen is prescribed. But those prescriptions are not all they seem (Webber 2010). It is exceedingly rare that theories based on individual consent prescribe the actual consent of citizens. They sometimes claim tacit consent (although the obstacles to refusing the acts that constitute tacit consent undermine that claim). Most commonly, they justify the constitution on the basis that the constitution’s provisions *would* or *should* or *could* be consented to, even if that “consent” can never be put to the test. The essential points are these. The nature and extent of consent is much less than generally claimed, indeed is much closer to acquiescence. That acquiescence is best understood to flow from the reasoning in Sect. 2.2 of this paper, namely the fact that settled institutions allow for peaceable collaboration and, in the case of democratic institutions, the testing of public support. It also helps that there is extensive experience with those institutions—that the institutions have a history in the society—for citizens then know how to engage in them, indeed have often internalized many of those terms, using them to frame their own positions even when they disagree on the specifics of those positions. Lastly, in democratic societies, the existence of stable mechanisms to amend those institutions means that, in principle, the citizens’ constitutional agency persists. They can revisit the previous decisions.

It is in all these senses that the constitution comes to be the people’s constitution. Note too that, with respect to citizens’ actual participation in creating or changing their constitutions, citizens typically act through institutions in which voting by majority, not unanimity, is the governing principle: constitutional conventions, legislatures, referenda. Even the decisions that certain processes be insulated from majoritarian pressures—the appointment of judges, the tenure of judges, the administration of elections—are typically adopted by majority decision. The public itself recognizes the value of insulating those matters from day-to-day majoritarian influence. The standard of legitimation for the system as a whole remains fundamentally democratic. Democratic legitimation is not contrary to the rule of law. It is the condition for the possibility of the rule of law.

The rule of law therefore depends upon democratic support to be effective. Several analysts have argued that, in the CEE, some post-communist constitutions were weaker than they ought to have been because those constitutions had been negotiated between dissident leaders and communist-era officials, with the outcomes enacted by the communist-era legislatures. They therefore lacked a sufficiently democratic imprimatur, or had impaired legitimacy because of the failure to dislodge communist-era elites (Arato 1993: 678–82; Blokker 2014; Czarnota 2016; Suteu 2019: 502; Jakab and Bodnár 2020: 106–7; Stambulski 2022: 351–9). One sympathizes with the people’s representatives. They accomplished an historic transformation in immensely challenging circumstances. But once successful, a more concerted attempt to bed down the constitutions democratically would have been desirable. Indeed, democracy involves continual work in assembling and re-assembling the public’s support. That is one essential aspect of what it means to have a people govern themselves continuously.

Finally, to be clear, in making this argument I do not mean to gloss over the flaws in democratic institutions or the evils that democracies can commit. Democratic institutions are imperfect because we, their people, are imperfect. The defects can be devastating. To take one manifest example, the very nature of citizenship means that the citizenry never includes all the residents of a nation’s territory. Above all, residents have often been excluded from political participation (or from life itself) on grounds of their racialized character, their gender, their Indigeneity, their ethnicity, their religion, or simply the deep unpopularity of some citizens’ opinions. Indeed, a central characteristic of right-wing populism is an insistence upon a limited definition of the people. Constitutional adjudication can go some distance towards remedying such defects, but one should not exaggerate the ability of courts to save us from ourselves. Courts too are staffed by imperfect humans, formed within their societies. They remain dependent on their societies’ political institutions for their staffing, funding, the carrying out of their orders, and the delimitation of their jurisdiction. It is a profound error to think that we as citizens can escape the requirement of our own democratic engagement. That is one of the lessons of Poland today, where the erosion of the rule of law appears to have been halted by democratic action. The rule of law is a continual democratic achievement.^10^

### Democracy and the Pursuit of Fidelity in the Application of the Law

Moreover, a central characteristic of the rule of law—indeed a definitional characteristic—is the faithful application of the law. This characteristic responds to Fuller’s eighth way in which a legal system can fail: “a failure of congruence between the rules as announced and their actual administration” (Fuller 1969: 39). But it also includes consistency between norm and application more broadly, for example: an ethic of fidelity in the interpretation of the law by both officials and judges; the exercise of public responsibilities in a good-faith attempt to fulfil public duties rather than achieve purely personal ends (thus forbidding corruption of all kinds); and doubtless other features.

All of these elements are advanced by the processes for maintaining and disclosing public records, fact-finding, oversight, accountability, parliamentary questioning and debate, and broader public freedoms of discussion and criticism that are fundamental to democracy.^11^ Rosanvallon (2006) has helpfully referred to this ensemble of measures as “contre-démocratie” with “contre” meant in the sense of counterpoint: interdependence, not opposition. These processes enable and support decisions made by democratic means. They are sustained by the legitimacy and authority held by members of representative bodies, tempering that of the executive. Indeed, democratic structures typically also serve consistency and congruence through the sheer formality of their articulation of the law. If nothing else, a statute provides a canonical statement of the law against which the conduct of officials can be judged. Indeed, democratic processes typically produce a host of materials that elaborate and explain the law: policy statements, guidelines, interpretation bulletins, reasons for decision, and the like. All these expose public action to scrutiny, testing, criticism, and justification.

Note, moreover, that processes of openness, scrutiny, criticism, and accountability are just as important with respect to courts as to the executive. The potential for criticism associated with democracy provides inducements to judges to act with integrity. It furnishes means for uncovering judges’ conduct when they do not. It is important to have special mechanisms for fact-finding in the case of judges given the constraints on judges’ ability to engage in public debate and their vulnerability to pressure from the executive and other actors, but nevertheless, without democracy, failures of judicial integrity would rarely be exposed. To take one contemporary example, the exposure by ProPublica (2023) of the undeclared gifts received by Clarence Thomas and Samuel Alito (including some from litigants before the US Supreme Court) serves, and does not detract from, integrity in the application of the law. One expects those gifts were known to members of the court; until exposure, no action was taken. Michał Stambulski’s example, in this special issue, of the Polish Constitutional Court’s decision in the printer’s case (Constitutional Tribunal K 16/17 of 26 June 2019) raises a less dramatic example. I imagine that very few observers, for or against that decision, believe that the reasons provided by the majority of the Court accurately expressed the majority’s motives. Public scrutiny allows commentators to test the reasons given, reveal instances in which judges are less than frank in the reasons they provide, and thus encourages (one hopes) greater openness and fidelity in the exercise of public duties. Moreover, public scrutiny and political freedoms support judges and other actors when they *do* resist the pressure to bend their judgements to the will of the powerful. Interference typically occurs behind closed doors. It can take courage and fortitude for a lone judge (or prosecutor or tribunal member) to resist. Public exposure—either the fact of exposure or its possibility—is often crucial in assisting those actors to act as they should.

More than 20 years ago, before the repressive turn under Xi Jinping, I attended a seminar in which a leading Chinese scholar of administrative law said that, in their view, China’s administrative law had reached its maximum development and that any further development would depend upon further democratic reforms. One can see why. Without such reforms, the capacity for disclosure, robust fact-finding, oversight, accountability, and criticism is substantially lacking. Historical accounts often treat the achievements of the rule of law and of democracy as though they were separate, with the rule of law said to predate democratic reforms. Perhaps. But, if so, it would have been a substantially truncated version of the rule of law.^12^

### Democracy and the Excellences of the Rule of Law

Finally, the existence of democratic government is crucial to the achievement of at least two of the eight excellences that Fuller ascribes to the rule of law.

#### Democracy and the Achievement of “General” Rules

The first is “generality”—that the law ought to take the form of general rules. This element is central to Fuller’s argument that the rule of law ought to respect citizens’ agency. He argues that under law, citizens are not ordered to perform certain actions in the same way that a manager might direct the actions of an employee. Instead, law consists of general rules, a general framework, within which citizens are empowered to make their own decisions (Rundle 2013: 127–9). Moreover, generality helps to ensure that government actions are driven by motives applicable to the citizenry as a whole, not by a desire to prefer the friends or punish the enemies of those in power. State action ought to be justified by public purposes and not driven by officials’ personal preferences and animosities. The requirement of generality therefore responds directly to one of the paradigmatic examples of disregard for the rule of law: bills of attainder, in which a legislature declares by statute that a named person is guilty of some crime. These two aspects of generality—(1) respect for citizens’ agency and (2) the insistence that public purposes, not private inclinations, should motivate state action—are bound together in the idea that the law should distinguish between, on the one hand, a private sphere that is subject to individual decision-making and, on the other, a public sphere that is subject to special requirements of public-spiritedness and impartiality.

The problem is how to distinguish between these two spheres or, to put the challenge in a different way, how to define precisely what constitutes a “general” rule. Bills of attainder may be a clear case, but once one goes beyond the case of named individuals, how does one distinguish between rules that are general and those that are not? A law’s generality cannot be reduced simply to the number of people to whom it applies. A law always attaches legal consequences to certain situations and not others, and that means that the law always applies to some people more than others. Even the protection of property, which is often foundational to theories that emphasize generality including those of Hayek (1944, 1960, 1973) and Oakeshott (1975, 1983), manifestly protects the interests of property-owners more than non-owners. The more property one has, the more one benefits. If one focuses only on the number of people affected by a law, generality will always be a matter of degree, and there clearly are acceptable rules that apply in practice only to very few people, even a single person, for example rules that govern the conduct of certain offices: in the private law the office of a trustee or tutor; in the public law, the President of a republic or the Director of Public Prosecutions. For that reason, theories that emphasize generality sometimes say merely that laws must apply to an *undetermined* number of people. That is helpful, but it still does not get to the heart of generality.

An especially influential answer to this conundrum was developed by Oakeshott (1975, 1983).^13^ His theory is, in important respects, similar to that of Hayek (1973) and I will therefore refer to both, but Oakeshott’s development of the position is by far the stronger. Oakeshott argues that general rules establish rules of social interaction without seeking to achieve particular outcomes. His position is best grasped by focusing upon his vision of the ideal relationship of citizens to their society. In this relationship, citizens do not join together to pursue common ends (to form, in his terms, a *universitas*) but rather to sustain an order of rules within which to pursue their own ends (thus forming a *societas*). Citizens in a *societas* are united only in their recognition of the framework of rules. Those rules are, in important respects, analogous to rules in games like chess, tennis, or cricket, in which the players compete purposefully within the game but the rules of the game itself are not aimed at achieving any purposes (Oakeshott 1983: 125–32). The law should aspire to that non-directive, non-purposive, generic quality. In Oakeshott’s theory, then, the generality of the law is tied to a limited role for the state.

There are, however, two conclusive objections to Oakeshott’s vision. One is that even in minimalist theories of the state, some of the things that a state must do are plainly aimed at achieving specific outcomes (Loughlin 2010: 331). But more fundamentally, it is simply unreal to suggest that the kind of association that Oakeshott has in mind—or really any association—avoids the privileging of particular ends. His preferred association was a highly specific one: the pursuit by individuals of their own objectives through the deployment of their property within a regime defined by general rules of contract, tort, and the criminal law. But that regime is hardly neutral as to the purposes of social life. Indeed, it was brought into being through the purposive action of law-makers who sought to achieve what were at the time contested social ends. Think, for example, of the substantial reforms to the law of property in land that abolished feudal tenures or, in the Common Law, drastically simplified those tenures to create something approaching full ownership of land. Those reforms are constitutive of the kind of *societas* Oakeshott has in mind, they substantially restructured the forms of property it was possible to hold, and they were driven by highly specific policy aims—such as, in the nineteenth century, the development and commercialization of land.^14^ Another example would be the destruction of Indigenous economies and legal orders, the dispossession of those peoples, the imposition of European forms of land tenure, and the reallocation of their lands to newcomers, which was similarly the product of a deliberate policy of social displacement and reconstruction (cf Waldron 2012: 28–31).

To put the point another way, there is no single game—no framework decreed by God or nature—that a society must adopt. There are several potential games and the choice among those games (I wager Oakeshott’s and Hayek’s own choices) is made on the basis of the purposes that the chosen game is understood to serve. It is only because Oakeshott brackets that choice and treats his game as the only game in town that he is able to maintain the fiction that his general rules are without purpose—or, as Hayek says of his similar theory, “purpose-independent”, “existing independently of anyone’s will” (Hayek 1973: I, 85). This kind of sleight of hand is precisely what critical scholars of all descriptions have attacked in their critique of legal doctrines that consign gender relations, or race relations, or the distribution of wealth to a private sphere beyond legal regulation; that treat the rules of contract law or tort law as merely the manifestation of a non-political corrective justice; or that treat property rights as natural when they were the product of deliberate construction. Indeed, the problems with Oakeshott’s and Hayek’s arguments are still more far-reaching, for they are only plausible at all if one remains at a level of high abstraction where no specific rights of any kind are analyzed but instead one refers only to something like “the conditions of civil association” (Oakeshott’s terms). But of course no such generic civil association exists. And when one does descend towards an actual association, all the messy purposes and intentions of real human beings begin to muddy the static and faux-harmonious waters of the theory.

Where does the failure of Oakeshott’s and Hayek’s projects leave the generality of the law? I suspect that the two features associated with generality—(1) respect for citizens’ agency and (2) insistence that public purposes must motivate state action—have real appeal for many theorists and certainly for many citizens. Even the deconstruction of the public/private distinction by critical theorists is, I believe, directed primarily against what those theorists consider to be the tendency of the law to treat that distinction as natural, necessary, and fixed—to place it beyond the reach of politics, to hide the fact that it is socially constructed—not against the construction of such a distinction in the first place. How then might the desirable features of generality be defined and secured?

A good first step is to realize that generality operates at the level of the *justification* for a law, not its breadth of application. The reason why a rule applicable to the office of the Attorney-General would be acceptable even though it might only apply to a single person is that the justification for that rule would apply to anyone filling such an office. General rules are general by virtue of the nature of their justification.

How then do we ensure that their justification has this generality? Many have turned to the theories of Rawls (1993, 1999) in his work on “public reason” and of Habermas (1996) in his attempt to specify the conditions of unforced agreement to determine rules that have a universally acceptable character. Those theories are sometimes interpreted as though they provide fully-determinate policies, so that a talented theorist, alone in their study, could reason through to the single acceptable outcome. That, I think, gets the theories wrong (or, to the extent it gets them right, Rawls and Habermas are wrong). First it presumes that those theories are adequate to provide single answers; there is a strong argument that such abstract theories are better designed for determining a range of acceptable outcomes. But second, even with respect to the determination of that range (and certainly when used to generate a single outcome), they underestimate the scope for legitimate disagreement in society. It shows considerable hubris to attempt, through theorizing alone, to determine the permissible scope of public debate, in effect determining what (and therefore who) is to be heard, what (and who) is to be excluded. Third, this is especially true when Rawls’ and Habermas’ theories depend upon questionable accounts of human reasoning: in the case of Rawls, the suggestion that positions grounded in a religious tradition or other “comprehensive doctrines” are incapable of persuading a non-believer (Rawls 1993: 212ff; contrast Stout 1988: 64ff; Nedelsky 2006; Webber 2012: 37–43); in the case of Habermas, the assumption that his explication of rationality identifies the only form of moral reasoning admissible (Taylor 1994: 246–8; 1998: 84–7; 2011: 1–15; DeSouza 1998; Tully 2008a: 71–131). Moreover, both seek to imagine a power-free arena of deliberation, but how can we imagine such a world given that we are finite humans, living in a particular time and place, always occupying a particular social position, raised in a particular language and culture? What does a generic human, living outside of all historical positioning, even look like? How do they judge what is good and what is bad? Surely, it is better to recognize our positioning and expose it to confrontation and revision, not pretend we have transcended it.

These theories are most appropriate for helping us to refine our contributions *within* public discussion, not establishing boundaries for that discussion and certainly not decreeing specific policies. Engaging with these and many other theories clarifies our options, but to come to legitimate outcomes in a world of diverse people, substantive theories must be complemented by a distinctively political theory of decision-making, one that takes the scope of citizens’ disagreement seriously, fosters fact-finding and accountability, and treats citizens as reasoning agents in their own right (Waldron 1999: 1–4). Legitimate outcomes are best determined by tolerating actual debate—not merely a notional debate—in an arena in which all those affected participate as nearly as possible as equals. It is best achieved by democracy.^15^

The breadth of participation provided by democracy speaks best to both aspects of generality identified above. It provides the best test of what policies are supported by genuinely public reasons because they are tested in as wide a public as possible (Arendt 1992: 42–44, 71–75; Nedelsky 2000: 249ff). It provides the best means of determining the limits within which individuals should be free to exercise their own judgement within a private realm precisely because democracy is founded on respect for citizens’ capacity to exercise judgement.

Democracy is designed to construct public outcomes in conditions of diversity. It does so in a manner in which citizens are entitled to participate on a basis of equality. The norm of equality may, in our unequal world, never be fully achieved, but at least it establishes an aspiration in relation to which access to participation can be judged. Democracy falls short, but it contains mechanisms for criticizing shortfalls and, if we take our task as citizens seriously, overcoming them. I suspect that, for every one of us, reality falls short of our particular hopes, but who would we nominate to take the place of the citizenry in exercising collective self-government? If not the citizenry, then whom?

#### Democracy and the Ability to Know the Law

The other form of excellence that is substantially dependent on democratic institutions is Fuller’s promulgation. Fuller develops his excellences in an allegory about a law-giving monarch (a different but related allegory from that discussed in this paper’s introduction). The phrasing of those excellences is especially appropriate to law-giving—to legislation—and is poorly adapted to other forms of law. I will therefore focus on the general value that underlies promulgation: that citizens must be able to know the law. This value informs several of Fuller’s excellences in addition to promulgation, including law’s clarity, constancy through time, and non-retroactivity. The rationale for all these excellences is that citizens need to know the law if they are to exercise agency within the law. The idea is simple but, as in generality, the simplicity hides real difficulty.

The first difficulty is that the requirement of promulgation does not apply at all well to judge-made law. Judge-made law is not promulgated in any conventional sense, but develops incrementally as decisions are made. Indeed, one of the classical reasons for displacing judge-made law through codification has been precisely to enable citizens to know the law (eg Bentham 1970 [1792]: 71, 152–4, 184–94). But second, even in the case of legislation (including codes), the sheer volume of the law is such that citizens can hardly be expected to know it all. How many renters of residential apartments have read the law governing their leases even when that law is codified (as it is even in many Common-Law jurisdictions) and even though those leases constitute a principal investment of many households (both financially and in terms of the households’ way of life)? Even if renters did read that law, could they be expected to anticipate the detail of its application? And of course, over time, the effective content of that law is rendered more precise through its application. Interpretation clarifies and supplements earlier statements of the law. Those interpretations occur in the very act of applying the law and are often crucial to the decision so that the parties to the specific proceeding have not been able to know in advance the law to which they are subject (cf Bentham 1970 [1792]: 190–1). Indeed, if that degree of knowledge truly were essential, the decision-maker should decline to apply the interpretation and should merely promulgate it for future cases, but of course that would run the risk of continual deferrals of the law’s application. One understands the exasperated practicality of article 4 of the French Civil Code: “Le juge qui refusera de juger, sous prétexte du silence, de l'obscurité ou de l'insuffisance de la loi, pourra être poursuivi comme coupable de déni de justice.”^16^ But if the conventional understanding of promulgation is right, isn’t that exactly what a conscientious judge should do?

Now, it is true that the purposes underlying promulgation extend beyond the ability of citizens simply to know the law. Promulgation helps to ensure that a supposed enactment has been duly authorized. It creates an authoritative text of the enactment. That text will be the starting-point for any application, all decision-makers working from the same text. Promulgation therefore serves another feature of the rule of law, namely that there be reasonable “congruence between official action and declared rule.” Nevertheless, most theorists of the rule of law, including Fuller, cling to the notion that the law ought to be knowable to its subjects in advance. That, after all, is a key element in Fuller’s argument that the rule of law respects the agency of those subject to the law. What sense can we make of it?

The answer begins, I think, with Gerald Postema’s emphasis (1994: 373–4) upon a type of congruence that is somewhat different from Fuller’s, namely “that legal norms and authoritative directives can guide self-directed social interaction only if they are broadly congruent with the practices and patterns of interaction extant in the society generally.”^17^ The idea here is that citizens’ knowledge of the law is not derived primarily from reading the law but from their participation in social interaction generally. Through that interaction they come to know the norms that govern various legal relations, perhaps in part because of the explicit legal knowledge they encounter but mainly from the norms they find embodied in the practices. These they learn implicitly as they engage in the practices. This legal knowledge is grasped more in outline than detail. If citizens anticipate trouble, they may take steps to inform themselves further. But at least they have a general sense of the operative norms. Indeed, I suspect that this is the method by which most people learn the law that governs their residential leases.^18^ On this view, then, the capacity to know the law is satisfied by the extent to which the official law is consistent with the understanding of law extant in society, not the reverse.

This interpretation of the principle fits well with the recognition that custom constitutes a source of law in both the Civil Law and the Common Law (although, in Postema’s conception, the role of custom extends much further than suggested by those limited doctrines). It also is consistent with a traditionalist vision of the Common Law in which the entire body of that law was once said to express the “custom of the realm” (Blackstone 1765: 67–73; Helmholz 2003), although see Bentham’s trenchant rebuttal of the notion that judge-made law is customary in any real sense (Waldron 1998: 100–8). But there is a further problem. Custom tends to be conservative, reproducing the “patterns of interaction extant in the society generally.” The benefit of those patterns—even the ability to participate in and know those patterns—is unevenly distributed. It is not as though courts have affirmed norms that command universal knowledge and assent. On the contrary, courts have tended disproportionately to express the interests of the powerful, of property, of those who can afford the courts, maintaining order above all, and historically treating many movements for broader participation in society (workers’ collective action, women’s equality) as disorder. Not uncommonly, aficionados of the Common Law—or, in Civil-Law traditions, defenders of the “general law” embodied in the Civil Code—have considered reforms secured through statute law to be inherently problematic because they depart from a supposedly consensual, supposedly rational, supposedly organic, perhaps even spontaneous, property-based order (see especially Hayek 1973: vols I and II). Indeed, Common-Law judges have sometimes shared this view, and have therefore shaped the Common Law precisely to undermine statutory reforms. The economic torts—still part of the Common Law in many jurisdictions—are a clear example (Orth 1991). The claim that the Common Law represents custom can, in other words, be partial in the interests it favours, biased in its articulation, static, and anti-democratic.

The slippage from recognition of custom to idealization of the Common Law to opposition to statutory reform is unfortunate. Postema’s “implicit law” does matter. Postema draws our attention to how most citizens come to know and deploy the law. If we want to improve citizens’ knowledge of the law—or even ensure that a reform to the law is fully implemented—we would be wise to turn our minds to it. Attention to customary law can also reveal abuses that would be invisible if we only focused on state law: eg metropolitan elites’ use of their differential access to state law to dispossess peoples whose rights are dependent on custom. Those abuses themselves have impacts upon rule-of-law values: they involve partiality and inequality in the administration of the law; they unsettle citizens’ expectations. Above all, the “implicit law” captures an indispensable ground of much legal reasoning. Any operative legal order is, to a substantial degree, customary (Webber 2009).

The problem lies in the uncritical idealization of the implicit law, namely the assumption that such law is single in its expression, power-free in its development, universally known, uncontested, adequately translated into the Common Law, even “extant within the society generally” in Postema’s sense (Webber 2006c). With respect to all these things, democratic engagement—both within the state and within non-state legal orders themselves—can increase the generality of both implicit and state law. Democratic processes make space for the expression of a wide range of viewpoints, including those that are not currently dominant. They provide authoritative and broadly-based means for settling contested customs. They provide avenues for political action, critique, reform, and (one hopes) the progressive inclusion of more members of society in the framing of law. Democratic action can produce better congruence in Postema’s sense, both in state law and in the implicit law itself, by broadening the range of voices empowered to speak to law.

Moreover, the prominence and participatory character of democratic processes directly promote wide knowledge of the law. This is obviously true of changes to the existing law as representatives consult, propose, debate, amend, and ultimately adopt proposals. Those deliberations themselves typically take place in the view of the press and sometimes the public but, in addition, the representatives themselves serve as a link between those decisions and their constituents, consulting, taking advice, reporting back, and ensuring that measures they support come to the attention of those who will benefit. The most important of those reforms may be foreshadowed in the campaigns that elected those representatives and cited in subsequent campaigns. The democratic process therefore contributes to the incorporation of revised norms into the implicit law, embedding those reforms within the society. Even without reform, democracy serves a role in disseminating knowledge of the existing law. Its functions of fact-finding, questioning, and demanding accountability publicize the content and administration of law.

### The Rule of Law Needs Democracy: Final Comments

As the state’s law becomes a product of democratic self-government and representatives of the citizenry become responsible for its articulation and development, citizens take hold of their law. Their role as authors of law makes for greater knowledge as addressees of law. These processes in turn shape how the law conforms to its people’s aspirations and is embedded in people’s understanding of law. Democracy builds congruence—though in this case, a dynamic congruence, changing through the participation of those who are both subject to and authors of their law. It is often precisely this inclusion of the citizenry, this broadening of their ability to debate and decide, that authoritarian populists seek to restrict through their projection of an artificial, restricted, backward-looking, anti-deliberative, disempowered, vision of their people.

## Conclusion

I began this paper by contesting the assumption that the rule of law is, fundamentally, about limiting democratic government. I argued that the rule of law is just as much about enabling democracy as it is about limiting. Democracy depends upon the processes by which a public position is formulated against a backdrop of disagreement, and the integrity of those processes depends upon the consistency and clarity associated with the rule of law. Democracy also requires that once decisions are made, they are faithfully implemented. Again, practices associated with the rule of law are essential. Of course, the rule of law is not all there is to discuss about democracy. It is one element in what should be a program to build democratic engagement, improve the structures that enable democratic decision-making, and ensure the adequacy and integrity of the mechanisms by which those decisions are carried into effect. It is also true that democracy is driven, fundamentally, by civic engagement among the public at large, not merely by institutional structures. That civic engagement has historically included, in times of crisis, action outside the law. But as I argued above, the merits of that recourse have to be considered carefully and, even in taking such action, it is wise to weigh fully the value of consistent, established mechanisms for making collective decisions both within social movements and in society at large. Democratic movements—movements that have remained democratic through to their conclusion—have typically done so. A healthy democratic society requires both civic engagement and institutional structures for clarifying and resolving (albeit provisionally) disagreement.

But it is a mistake to treat the relationship between the rule of law and democracy as though it only operated in one direction. The two principles are interdependent. This was true historically. The development of the rule of law occurred in tandem with and was supported by the expansion of political participation. It remains true today. The institutions of the rule of law are dependent upon popular support for their legitimation. They function best when they benefit from the transparency, oversight, criticism, accountability, and testing of information associated with democracy. Moreover, democratic processes provide the substance for key dimensions of the rule of law, especially the progressive generalization of the law’s justification, and congruence between the state’s law and citizens’ understanding of law.

Democracy and the rule of law are, then, mutually dependent and mutually reinforcing. They arose together and they flourish together. The erosion of one impairs the other. We see that interrelationship in the populist regimes of the CEE. The governments of undemocratic societies might claim legal authority but their legal institutions, like their representative bodies, are compromised and truncated. Their time in government has weakened the rule of law. It has degraded their countries’ democratic practices. Their countries are both less democratic and less secure in the administration of the law.

This interdependence of the principles holds important lessons for how one ought to respond to authoritarian populist governments. Such governments are unlikely to be controlled effectively by legal institutions alone, at least in the long term. There is little alternative but to contest those governments’ claims in the political arena. That contestation may fail. Authoritarian regimes of all kinds do their best to insulate themselves from democratic change by restricting the release of information, limiting debate, monopolizing or hobbling the press, harassing opponents and NGOs, and impairing the independence of the electoral apparatus itself. Indeed, the Orbán regime, with its super-majority, has sought to protect some of its policies even against future democratic majorities by enshrining them in the Hungarian constitution or in cardinal laws (Suteu’s paper in this special issue; Suteu 2021; Venice Commission 2011). There could not be a clearer demonstration of their disdain for the agency of the actual people of Hungary. But there are limits to the extent to which populist governments can suppress democratic accountability without forfeiting, for all to see, their claims to popular legitimacy. It is crucial that one continue to press for that accountability so that the erosion is visible and the mechanisms that remain preserve the vigour they still have.

It is also important that both domestic parties and the international community advocate for democratic reforms, not just the better enforcement of law, whether authoritarian populists are in power or not. That advocacy ought to include mechanisms for public accountability, guarantees for parliamentary debate and deliberation, measures against corruption, and the preservation of robust electoral machinery. But in addition, one should not neglect the material underpinnings of a healthy democracy. In that regard, the trend towards greater economic inequality has been corrosive, undermining many citizens’ trust that their country’s institutions work for them, thereby triggering populist reaction (Webber 2023: 864–5, 870–2). Reformers would be wise to rebuild that crucial foundation of a vibrant democratic culture.

I suspect that many readers might see those prescriptions as thin gruel, given that they depend upon the citizens’ exercise of democratic freedoms within the system itself. Many long for solutions that come from outside the system—solutions that can ensure governments simply do what they ought to do and prevent mistakes that citizens might otherwise make. The hope for a *deus ex machina* is precisely why the language of limits is so common in the rule-of-law literature. But it is always, everywhere, a mistake. Like it or not, the fact is that humans are responsible for their own governing institutions. That institutional structure is self-regulating or it is not regulated at all. Courts in particular are not outside of that structure; they too are staffed by humans, chosen by other humans, funded in some fashion, and make orders that must be respected or enforced by other humans, all within the governing structure. We have no choice, then, but to rely upon self-regulating mechanisms. That does not mean that every part of the institutional matrix must operate in the same way. On the contrary, a blending of institutional forms is likely to produce the best results. But it does summon constitutional lawyers back to their true calling: designing institutional structures that build on mechanisms’ strengths and compensate for their weaknesses, that maximize the likelihood of virtuous circles and minimize vicious ones. We should not hold out for some external authority, beyond the reach of self-government, that will get everything more right than we can attain for ourselves.

So what is the alternative to the language of limits? I think that the best alternative may be the *discipline* of the rule of law—a discipline that is predominantly developed internally. In recent work, Martin Krygier (2017, 2019) has referred to the rule of law as “tempering” power. That term too strikes me as appropriate in its move away from the language of external limitation.^19^ The idea of a self-imposed discipline is surprisingly consistent with the classical works on the rule of law in the British, German, and French traditions. Consider, for example, Dicey’s advocacy of both the rule of law and parliamentary sovereignty as the twin, compatible, principles at the foundation of the British constitution (Dicey 1959). Moreover, that notion of discipline keeps our gaze firmly focused on our responsibility as citizens. Lon Fuller describes his eight features as “excellences”. He does so because they are not merely a checklist of prohibitions but demand judgement and imagination in their application (Rundle 2013: 91–2). They require positive as well as negative actions. That necessity for judgement is precisely why Waldron (1999) argues for the primacy of democratic decision-making.

This way of conceiving of the relationship between the rule of law and democracy builds upon a principle that has become evident at many points in this paper, namely the interdependence of the statuses of citizens as the authors and addressees of law. Many contemporary treatments of the rule of law affirm that the rule of law is founded upon respect for citizens’ agency: upon the value of citizens being able to know the law and to arrange their affairs in reliance upon that knowledge. In so doing, those discussions rightly recognize the dignity of citizens as addressees of law. But often, at the very same time, they sharply separate this dignity from respect for citizens as active, self-determining authors of law. On this other side of the coin, they demonstrate considerable distrust for citizens’ agency, arguing for a substantive rule of law, pre-defined by the theorist (Habermas? Rawls? Hayek?) and destined to be enforced by courts, as a necessary restriction on self-government. It is almost as though we have projected onto our twenty-first century democracies the misgivings of those nineteenth century liberals who, at the early stages of extension of the electoral franchise, worried that the “tyranny of the majority” exercised by newly enfranchised workers, might encroach upon the sanctity of property.^20^ Those fears weren’t justified. (I wish they were: we could use more encroachment given the growth in inequality in recent decades.) The fears expressed in today’s literature tend to focus on the rights of other minorities than the rich. But it is worth asking whether those fears are, in the case of democracies, just as unjustified—indeed more so given that the very instruments that are used to protect rights were invariably created by democracies themselves. And we should also ask whether, in their anxiety to impose limitations, those theorists will, like the propertied liberals before them, over-limit and over-define, impairing the agency of all citizens. It is striking that some of today’s most important work on remedies for human rights violations emphasizes the value of participatory remedies, in which disadvantaged citizens are able to join together with their advantaged counterparts more equally and effectively to fashion their future relations (Sheppard 2010; Liebenberg 2015; Deveaux 2021).

The fact is that citizens are both authors and addressees of law. Those statuses are not separated by the great gulf postulated by much of the rule-of-law literature. They are interdependent and mutually-reinforcing, imposing compound responsibilities. We are fated both to live in society and to develop our own understanding of the aims in life that are most important. Those tasks exist together. We may, of course, fail at them. But the important fact about democracy is that success or failure lies in our hands.

## Footnotes

1 For the concept and implications of populism, see Webber (2023).

2 For a useful categorization of “thick” and “thin” definitions of the rule of law, see (Møller and Skaaning 2014: 13–27). This paper’s approach is similar to that of the Council of Europe’s European Commission for Democracy through Law (the Venice Commission), in which human rights, democracy, and the rule of law are treated as analytically separate but interdependent (https://www.venice.coe.int/WebForms/pages/?p=01_Presentation&lang=EN). See also *Declaration of the High-level Meeting of the General Assembly on the Rule of Law at the National and International Levels*, GA Res 67/1, UN GAOR, 67th Sess, UN Doc A/RES/67/1 (2012) para 5: We reaffirm that human rights, the rule of law and democracy are interlinked and mutually reinforcing and that they belong to the universal and indivisible core values and principles of the United Nations. The range of content ascribed to both democracy and the rule of law by other international actors can be very broad. The definition of the rule of law by the influential World Justice Project (https://worldjusticeproject.org/) includes four “universal principles,” with part of the second being that the law “ensures human rights as well as property, contract, and procedural rights.” Interestingly, the index used by the WJP to measure the rule of law includes a substantial list of first-generation rights and freedoms but not property or contract, except to require “lawful process” and “adequate compensation” in expropriation (World Justice Project 2023: 14–19).

3 The rest of this paper will speak of state-structured societies but I hope that, in its essentials, it is responsive to non-state forms of social organization. For descriptions of the governing institutions of one such people, the Gitxsan of northern British Columbia, see (Daly 2005: 66–96, 290–4; Napoleon 2010, 2022; Sterritt et al. 1998: 11–13; Webber 2022) For emphasis upon the breadth of potential forms of democracy generally, see, among many others, Tully et al. (2022).

4 The criticisms of “rule by law” themselves suffer from ambiguity over what is meant by that phrase. Indeed, one gets the impression that separate “traditions”— Meierhenrich (2021)’s term (at 47)—of “rule by law”, “rule of law”, “rule under law” have a clearer definition for today’s theorists than they do for those whose views are being described. In particular, some theorists treat “rule by law” as requiring nothing more than some form of state authorization. If that is one’s definition—if one gives no credence to the formal qualities of law developed below—then it may be true that rule by law involves no worthwhile discipline (Møller and Skaaning 2014: 17; this may also be Loughlin’s position (2010: 332–5)). In my view, that is usually a misreading of the way in which “rule by law” has been used. Loughlin (2010: 314–24) provides a valuable overview of the literature on rule of law and its counterparts in the British, German, and French traditions, noting that that phrase has often been used in the sense of “rule by law”. In my view, the vast bulk of that literature turns on claims about the distinctive form of law. Of course, many theorists consider that the rule of law must include an array of substantive requirements because otherwise legislatures can pursue bad ends through law. It is true that democracies can do bad things, as indeed can bad judges. But such an expanded definition should be resisted for the reasons mentioned in the text, namely that it occludes or prejudges important institutional questions. This is especially the case when principles of natural law are read into the rule of law and then assumed to be enforceable by courts. It is true that, in 17th-century England (and at many other times), natural law was invoked as an important restriction on how power should be exercised, but judges were not considered the only font of that law. On the contrary, Parliament was considered at least as much an interpreter of the natural law as the courts. To see how the institutional consequences can get out of hand, consider the list of restrictions that Tamanaha (2004: 96) reads into the “rule of law”: “natural law, shared customs, Christian morality, or the good of the community.”

5 The discussion of co-originality does, however, run into the conceptual overlaps and vagaries of this area. Just what two things are co-original? Some articulations say democracy and human rights (eg Habermas 1996: 454, 2001: 767), others democracy and constitutionalism (eg Tully 2008b: 92–3), and still others democracy and the rule of law.

6 As the notes make clear, my account combines lessons from three of Fuller’s allegories, all variations on a similar story. The predominant allegory cited here (from Fuller 1968) concerns a ruler who appears to be using what Fuller would consider “managerial direction” rather than law properly so-called. My account slips from that story to a lesson about law. For the limited purposes of this argument, that conflation poses no problems. For an illuminating discussion of the 1968 allegory, see Rundle (2013: 108–12).

7 I use the term “citizen” throughout this paper both for those subject to law and those entitled to participate in the democratic life of their society. The term is imperfect especially when describing those subject to law, for of course the law applies to many parties who are not citizens, such as non-citizen immigrants and other residents, visitors, and corporations. I don’t mean to foreclose careful scrutiny of the status of citizen. I use the term simply to avoid too cumbersome a text. That term does capture a substantial proportion of the residents of a democracy. It also draws our attention to the relationship between citizens as authors and citizens as addressees of law. Nevertheless, there is much that ought to be debated about the attribution and regulation of that status.

8 Such an emphasis upon validity is characteristic of legal positivism. Indeed, Tom Campbell’s “ethical positivism” is founded on the obligation of judges to focus on validity precisely in order to respect democratic decision-making (Campbell 1996). Legal positivism combines this focus with an untenable theory of legal interpretation. However, it is possible to incorporate a respect for democratic decision-making within more satisfying interpretive theories, where it takes the form of an ethical commitment conditioning interpretation (Webber 2000, 2007).

9 One sees this elision in Meierhenrich (2021). He begins his discussion of the rule of law in England by saying that “law was widely regarded as a check on—not channel of—sovereign power” (46) and, as he proceeds, it is clear he is claiming that, in this tradition, law is understood as a check on all sovereign power including parliament. Yet he uses that phrase to introduce a quotation from Zweigert and Kötz (1998: 195) that begins: “The Common Law became a mighty weapon in the hands of the *Parliamentary* party in the struggle against the absolutist prerogatives of the King…” (my italics). Surely the elision requires more thought. Indeed, one cannot help wondering, even on Meierhenrich’s own account, whether the determining difference between rule of law and *Rechtsstaat* is not the role of natural law but the relative presence or absence of a democratic ethos.

10 The contributions of the Polish/Australian constitutionalist and theorist, Wojciech Sadurski, exemplify this development. His first book on populism and constitutionalism in Poland (Sadurski 2019) focused predominantly on the threat to constitutional adjudication (although chapter 5 of that book also chronicled the erosion of democratic institutions). His more recent book (Sadurski 2022)—and his extensive public engagement—has focused upon democratic action in support of both democracy and the rule of law.

11 The tendency of democracy to counter one of the principal features of neglect for the rule of law—political corruption—is supported by empirical studies (see, eg, Adserà, Boix, and Payne 2003; Escresa and Picci 2020). Note the importance of including measures of Rosanvallon’s *contre-démocratie* alongside measures of electoral competition.

12 The literature on Nazi law furnishes additional cases that bear upon this point. Much of the jurisprudential debate has focused on whether the enactments of such a vile regime should be considered “law” given their lack of conformity to basic principles of substantive justice. That question is important, but the cases also raise the issue of whether law, in any recognizable sense, was operative at all. Given the utter lack of accountability, given the frequent resort to extra-legal coercion, there were no constraints upon, indeed every inducement encouraging, judges and other state actors adapting their decisions to whatever individuals in power might prefer. In what sense, then, were judges enforcing a law of the state? See Dyzenhaus (2008): especially 1011–21.

13 See the valuable summary in Loughlin (2010: 324–30).

14 The purposes that drove those changes are most apparent when feudal forms of property were transformed wholesale by legislation. See, with respect to the purposes of the Code Napoléon’s provisions on property, Portalis (1836) and, with respect to the abolition of seigneurial tenures in Quebec, Young (1986: xii-xvi, 88–107). The purposes are more difficult to encapsulate in the Common Law because the changes occurred in bursts of activity scattered over a long period of time, but note that this was not simply the kind of incremental decision-making by judges that Hayek praises. Major shifts were accomplished by the Norman Conquest itself and then by legislation. Here is a partial list of such changes in England alone (in roughly chronological order): eliminating allodial landholding to require that all land be held ultimately of the king, abolishing subinfeudation, reducing feudal incidents, imposing controls on mortmain, permitting and then regulating testamentary disposition, reducing the number of tenures, abolishing the monasteries in order to convert their lands to non-religious ownership, enclosing commons, abolishing dower and curtesy, enabling married women to exercise their own property rights, radically simplifying English land law in 1925, prohibiting new entails (Baker 2019: 241–315, 523–6). Hayek’s idealization of the Common Law as a law developed only “by the independent courts” and “a law existing independently of anyone’s will” (not even the will of the courts?) is therefore entirely without foundation (1973: I, 85). Such a suggestion might be forgivable when applied to contracts or torts; it is plainly wrong when applied to the structure of rights in land. Oakeshott, on the other hand, acknowledges the necessary role of legislation (eg 1975: 138–41) but in doing so he struggles mightily to maintain that the law ought to remain unconcerned with purposes. He says that legislation is needed to respond to “notable changes of belief or sentiment about the desirable conditions of civil conduct” (138) but still seeks to draw a distinction between that “desirability” and the pursuit of instrumental purposes. He wants to maintain a hard distinction in a context that seems only to involve gradation: in which purposes certainly are present, and the only question is whether those purposes operate at a sufficient level of generality that citizens retain a sufficient margin of action. For a closely related exercise in Oakeshottian hair-splitting, see Loughlin’s discussion of Oakeshott’s concept of the *jus* of *lex* (2010: 331–2). Hayek (1973: I, 88–89) concedes that legislation may be needed to correct judge-made law, but in his case the concession is so out of keeping with the rest of his argument (especially his root and branch criticisms of legislation (1973: vols I, 124ff and II) as to raise doubt about its sincerity.

15 Rawls (1993) goes a significant distance towards this democratic outcome in his embrace of the “burdens of judgment” and his adoption of “overlapping consensus” as the foundation for public reason, but he nevertheless seeks to prescribe that consensus’s content and to pose acceptance of that content as the price of admission to political argument. This raises several problems. Consider three: (1) what is meant by consensus and to what range of decisions does it apply? Is this essentially a requirement of consent so that the legitimacy of the political order is determined by a requirement that all far-reaching political decisions be acceptable to essentially all citizens, or does such a system permit majority determination of such issues? For example, is it acceptable for a democratic society to engage in redistributive taxation in order to achieve a more equal society even if those at the upper end of the income distribution oppose it? If the answer is no, isn’t the resulting income distribution just as much the result of a contested social decision (in this case, the veto against redistribution) as a decision in favour of redistribution would be? (2) Rawls treats the overlapping consensus as defining a sphere of public reason that can then serve as a set of principles, separate from citizens’ actual commitments, which can be used, self-sufficiently, to determine any public issue. But are citizens’ principled commitments capable of being compartmentalized in that way? And how do citizens even know the boundaries of what is permissible, given that public reason cannot be static but continually evolving and in any case is specified only at a level of generality? If citizens can accomplish such a compartmentalization, can this be achieved without (a) substantially disenfranchising some citizens, or (b) forcing them to hide their true motives and instead provide surrogates? (3) In what sense is Rawls’ overlapping consensus a consensus at all if (a) it is determined by the theorist in advance and (b) then used to determine which arguments are admissible? I suspect that all democrats hope that broad agreement might be achievable on at least some foundational issues. The idea, then, of an overlapping consensus is attractive. But surely it needs to be continually sought *within* democratic deliberation, not imposed from outside. Rawls wants to establish conditions in advance in order to ensure that the system is stable, but given the gravity of the consequences (the exclusion of policies from democratic decision; the exclusion of arguments; the inducements to engage in insincere arguments; perhaps, as a consequence, the effective exclusion of people), the judgement with respect to stability had better be right. There is some suggestion in Rawls (1999) that the specification of public reason is rudimentary (accepting merely “a constitutional democratic regime and its companion idea of legitimate law” (132)) and, beyond that, operates essentially as an ethic of democratic engagement for citizens *within* a democratic political order. To the extent that is so, there is very little distinction from the position for which I argue.

16 “The judge who shall refuse to determine under pretext of the silence, obscurity, or insufficiency of the law, shall be liable to be proceeded against as guilty of a refusal of justice.” Translation from *Code Napoleon; or, The French Civil Code. Literally Translated from the Original and Official Edition, Published at Paris, in 1804. By a Barrister of the Inner Temple* (London: William Benning, 1827), online: https://www.napoleon-series.org/research/government/code/book1/c_preliminary.html.

17 Fuller (1969: 49–50) does approach this insight in his discussion of how to understand promulgation in the context of the Common Law, suggesting that the lack of strict promulgation need not be a problem as long as the Common Law tracks normative conceptions in the society at large. See also Fuller (1969: 64) in relation to clarity: “Sometimes the best way to achieve clarity is to take advantage of, and to incorporate into the law, common sense standards of judgment that have grown up in the ordinary life lived outside legislative halls.”

18 The North American access-to-justice literature makes clear that, even when citizens experience a dispute, they commonly turn to informal sources of legal information more than they do to lawyers or other formal sources (Institute for the Advancement of the American Legal System 2021: 102–5; Gower 2024: 27, 36–7).

19 Krygier retains “limiting” as a second dimension of the rule of law. I wonder whether, in his mind, that dimension is more associated with the executive than the legislature—whether it is, in other words, a function of the less democratic character of the executive.

20 Concern for the sanctity of property still lies uncomfortably at the foundation of some of today’s rule-of-law literature. For a convincing response, see Waldron (2012). One aspect is Hayek’s continued role, for many theorists, as a treasured interpreter of the rule of law. It is patent that Hayek’s principal purpose was to defend a small-state, property-based libertarianism against legislation aimed at achieving greater equality. I expect (hope?) that some of these theorists reject Hayek’s libertarian interpretation, yet there is real question, too seldom asked, of whether those aspects on which they rely can be separated from those they reject. After all, Hayek’s aspiration of a law beyond human purposes was intrinsically tied to his propertied individualism. What is left if that is rejected, and what does rejection itself say about the aspiration to a beyond-dispute vision of the appropriate domain of law? There is much to be said for Moyn’s caution (2019) that human rights adjudication has frequently been used to protect privileged minorities *against* equality, and Waldron’s (2012: 90–4, 105–7) and Peirone’s (2021: 230–1) reminder that the rule of law is routinely invoked to protect transnational companies against regulation by developing countries.
